# Temperature effect on coagulation function in mild hypothermic patients undergoing thoracic surgeries: thromboelastography (TEG) versus standard tests

**DOI:** 10.1186/s13741-024-00405-8

**Published:** 2024-06-12

**Authors:** Shangyi Hui, Qian Zhang, Jiaxin Lang, Jie Yi

**Affiliations:** 1 Department of Anesthesiology, Peking Union Medical College Hospital, No. 1 Shuai Fu Yuan, Wang Fu Jing Street, Beijing, 100730 China; 2 Department of Anesthesiology, Hebei Petrochina Central Hospital, No. 51 Xin Kai Road, Langfang, 065000 China

**Keywords:** Mild hypothermia, Coagulation function, Thromboelastography, Standard tests, Thoracic surgery

## Abstract

**Purpose:**

Our previous research has revealed that mild hypothermia leads to excessive bleeding in thoracic surgeries, while the underlying mechanism stayed unrevealed by the standard coagulation tests. The research question in this study was as follows: “How does mild hypothermia impair the hemostatic function in patients receiving thoracic surgeries?”. The purpose was to detect the disturbed coagulation processes by comparing the TEG parameters in patients receiving active *vs*. passive warming during thoracic surgeries.

**Methods:**

Standard coagulation tests and thromboelastography (TEG) were adopted to compare the hemostatic functions in patients receiving active *vs*. passive warming during thoracic surgeries. Furthermore, blood samples from passive warming group were retested for TEG at actual core body temperatures.

**Results:**

Sixty-four eligible patients were included in this study. TEG revealed that mild hypothermia significantly disturbed coagulation by decreasing MA (59.4 ± 4.5 mm vs. 64.2 ± 5.7 mm, *p* = 0.04) and α angle (70.4 ± 5.2° vs. 74.9 ± 4.4°, *p* = 0.05) and prolonging ACT (122.2 ± 19.3 s vs. 117.3 ± 15.2 s, *p* = 0.01) and K time (1.9 ± 1.0 s vs. 1.3 ± 0.4 min, *p* = 0.02). TEGs conducted under core body temperatures revealed more impaired coagulation than those incubated at 37 °C. Furthermore, postoperative shivering and waking time were significantly increased in mild hypothermic patients.

**Conclusion:**

Mild hypothermia significantly impaired coagulation function in patients receiving thoracic surgeries, which could be detected by TEGs other than the standard coagulation tests. Temperature-adjusted TEGs may provide a preferable method of hemostatic monitoring and transfusion guidance in thoracic surgeries, which warrants further clinical investigations.

## Introduction

Perioperative core hypothermia commonly occurs in patients receiving thoracic surgeries under general anesthesia (Yi et al. 2015; Yi et al. 2017). Apart from the impaired thermoregulation induced by general anesthesia (Leslie and Sessler 2003), direct exposure of the surgical field to the ambient temperature (Matika et al. 2017) and the prolonged operation duration in thoracic surgeries jointly exacerbate the heat loss (Kim 2019; Mori et al. 2019). Core hypothermia increases postoperative complications, including shivering (Kurz et al. 1995), delayed waking from anesthesia (Lenhardt et al. 1997), and excessive bleeding (Schmied et al. 1996). Severe hypothermia (core body temperature < 33 °C) has been recognized as causative of perioperative coagulopathy with robust evidence from previous researches (Ruzicka et al. 2012; Polderman 2012). However, lesser attention has been paid to the mild hypothermia (core body temperature of 34–36 °C) on its effect on disturbing the coagulation function in thoracic surgeries.

Our previous clinical investigation has revealed that mild hypothermia leads to excessive bleeding in patients receiving thoracic surgeries (Yi et al. 2018). However, the underlying mechanism of the hemostatic disorder was unrevealed by the standard coagulation tests (prothrombin time, PT; activated partial thromboplastin time, APTT; international normalized ratio, INR; fibrinogen level, FBG; platelet count, PLT).

Herein, we hypothesize that mild hypothermia significantly impairs the intrinsic coagulation function in patients receiving major thoracic surgeries. Besides, the impairment may not be detectable by the standard tests performed on blood samples warmed to 37 °C. Therefore, we applied standard coagulation tests and thromboelastography (TEG) to compare the hemostatic functions in patients receiving active *vs*. passive warming during thoracic surgeries. Furthermore, blood samples from passive warming group were retested for TEG at actual core body temperatures. Clinical outcomes, including postoperative shivering occurrence and waking time, were also reported. This is the first study conducted to explore the effect of mild hypothermia on patients’ coagulation function in thoracic surgeries and to investigate the potential advantage of TEG in quantifying hemostatic disturbances in such clinical scenarios.

## Materials and methods

### Participants and trial design

In this prospective, parallel two-armed randomized controlled study, we compared the standard coagulation tests and thromboelastography parameters, postoperative waking time, and shivering occurrence in active *vs*. passive cutaneous warming patients with the core body temperature as the primary endpoint. It was an open-label study due to the nature of intervention. To be eligible for enrollment, patients must be ≥ 18 years of age, scheduled for elective radical resection of pulmonary cancer/esophagus cancer (operation duration > 2 h), and with core body temperature between 36 and 37.5 ℃ prior to surgeries. Exclusion criteria included the following: patients under 18 years of age, hemoglobin level (HB) ≤ 10.0 g/dl, platelet count < 100 × 10^9^/l or > 400 × 10^9^/l, prothrombin time (PT) prolongation > 3 s, activated partial thromboplastin time (APTT) prolongation > 10 s, international normalized ratio (INR) > 1.5, history of thyroid dysfunction, coagulopathy, intake of anticoagulants/NSAIDs within 14 days prior to surgery, or fever with infectious causes in the preceding 4 weeks. Demographic data and clinical data were prospectively collected since enrollment.

Enrolled patients were randomized into the passive cutaneous warming system (Control) and active cutaneous warming systems (Intervention) groups. Randomized numbers were generated by SAS 9.0 (SAS Institute, Cary, NC, USA) and sealed in envelopes. Anesthesia was induced by intravenous infusion of 1–2 mg/kg of propofol, 0.25–0.3 µg of sufentanil, and 0.6 mg/kg of rocuronium and was maintained by continuous intravenous infusion of 3–6 mg/kg/h of propofol, 0.1–0.3 µg/kg/min of remifentanil, and 0.75–0.1 mg/kg of rocuronium. Bispectral index (BIS) level was monitored between 40 and 60 during the surgeries. Patients in the Control group were intraoperatively covered with unwarmed surgical draping, while patients in the Intervention group were preoperatively and intraoperatively warmed to 37.5 °C core body temperature with the Bair Hugger warming unit (Bair-Hugger Patient Warmer, 3 M, St. Paul, MN, USA) (Alparslan et al. 2018). Tympanic membrane temperature was measured as core temperature at 5 min (min) before anesthesia induction (T1). Nasopharyngeal temperature was measured as core temperature 1 h after operation initiation (T2) and until the completion of surgery (T3). Once a core temperature below 36 °C was detected in the Control group, a forced-air patient warming system (WarmTouch Patient Warming System, Covidien LLC, USA) would be applied as rescue remedy, to restore the core temperature above 36 °C. One heparin-free arterial catheter was inserted into a radial artery for invasive blood pressure monitoring and blood sampling in each patient.

This study was approved by the scientific ethical committee of Peking Union Medical College Hospital (PUMCH) and registered at ClinicalTrials.gov (NCT03878901). Written informed consent was obtained from all patients before enrollment.

### *Standard* c*oagulation* t*ests*

**Fig. 1:**
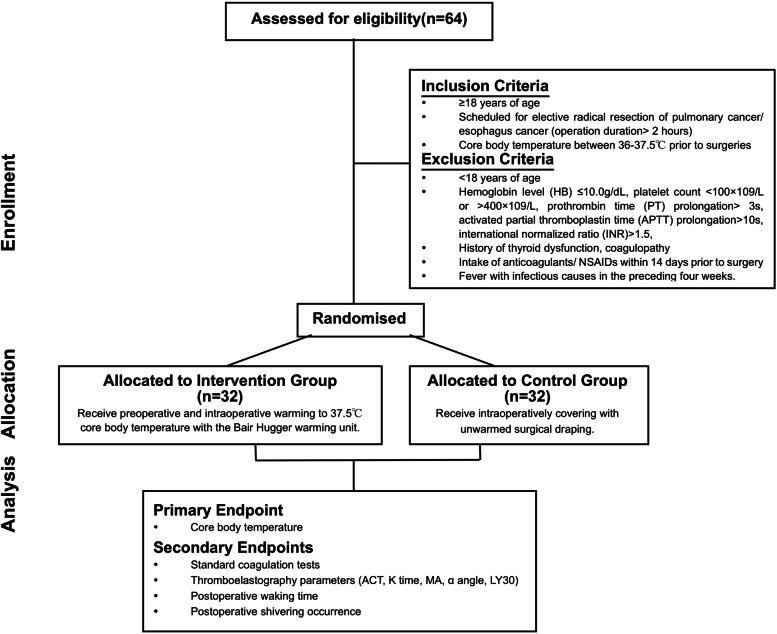
Flow diagram of the trial progress through the phases of enrollment, intervention allocation, and data analysis

Baseline coagulation function was examined using standard tests at enrollment. At surgical closure, 4 ml (ml) of arterial blood samples was collected in silicone-coated tubes containing 0.129-M buffered sodium citrate and sent for examination at the institutional lab. Five quantitative measures (PT, APTT, INR, FBG, PLT count) were recorded and compared between the two groups (Fig. 1).

### Thromboelastography

Coagulation functions at baseline and surgical closure were tested using TEG. Two temperature-adaptable TEGs® were used in this investigation. One was adjusted to 37 °C, while the other was adjusted to the actual core body temperature measured in the Control group. The temperature accuracy was verified by measuring the temperature of the thromboelastography cuvettes filled with normal saline and was within a deviation of ± 0.2 °C. Measurements were performed with disposable plastic pins and cups (Haemoscope), inserted at least 20 min before measurements to confirm the exact temperature of the cup. Immediately after sample extractions, 360-µl blood samples from the Intervention group were tested at 37 °C. Dual 360-µl samples from the Control group were concurrently tested at 37 °C and adjusted temperatures.

### Statistical analyses

Standard coagulation tests, TEG parameters, and waking time after anesthesia were presented as mean ± standard deviation (SD) and compared between groups using one-way ANOVA analysis. Incidence of postoperative shivering was enumerated and compared between groups using the chi-square test. Statistical analyses were performed using SPSS 19.0 software, and a *p*-value ≤ 0.05 was considered statistically significant.

## Results

### Study population characteristics

Sixty-four eligible patients (43 male, 21 female) were enrolled in the study. Patients were scheduled for video-assisted thoracoscopy (VATS) or radical surgeries for esophageal carcinoma. The demographic data, anesthesia and operation durations, intraoperative blood losses, infusion, and infiltration fluids were comparable between groups. Complete standard coagulation and TEG tests were conducted prior to surgeries, revealing no significant difference between Control and Intervention groups at baseline (Table 1).

**Table 1:** No significant difference was detected between Control and Intervention groups at baseline

Variables	Passive warming group (Control group)	Active warming group (Intervention group)
**Demographic data**		
*Gender (male/female)*	24/8	19/13
*Age (years)*	61 ± 11	59 ± 9
*BMI (kg/m*^*2*^*)*	23.2 ± 2.7	24.0 ± 2.6
*Intraoperative blood loss (ml)*	281 ± 167	296 ± 173
*Intraoperative IV fluid (ml)*	2239 ± 1094	2398 ± 961
*Intraoperative irrigation fluid (ml)*	2041 ± 1010	2509 ± 1341
*Anesthesia duration (h)*	4.4 ± 1.8	4.4 ± 1.6
*Operation duration (h)*	4.0 ± 1.7	4.1 ± 1.6
**Baseline routine coagulation test**		
*PT (s)*	11.4 ± 0.8	11.2 ± 0.6
*APTT (s)*	26.0 ± 2.8	26.4 ± 2.8
*INR*	1.0 ± 0.1	1.0 ± 0.1
*FBG (g/L)*	3.0 ± 0.9	3.4 ± 0.9
*PLT (*× *10*^*9*^*/l)*	201.2 ± 50.8	215.6 ± 53.8
**Baseline TEG tests**		
*ACT (s)*	113.1 ± 14.3	113.8 ± 16.8
*K time (min)*	1.3 ± 0.4	1.4 ± 1.2
*α angle (°)*	74.2 ± 3.2	75.6 ± 2.8
*MA (mm)*	62.8 ± 5.4	65.0 ± 4.9

### Changes in core body temperatures

**Fig. 2:**
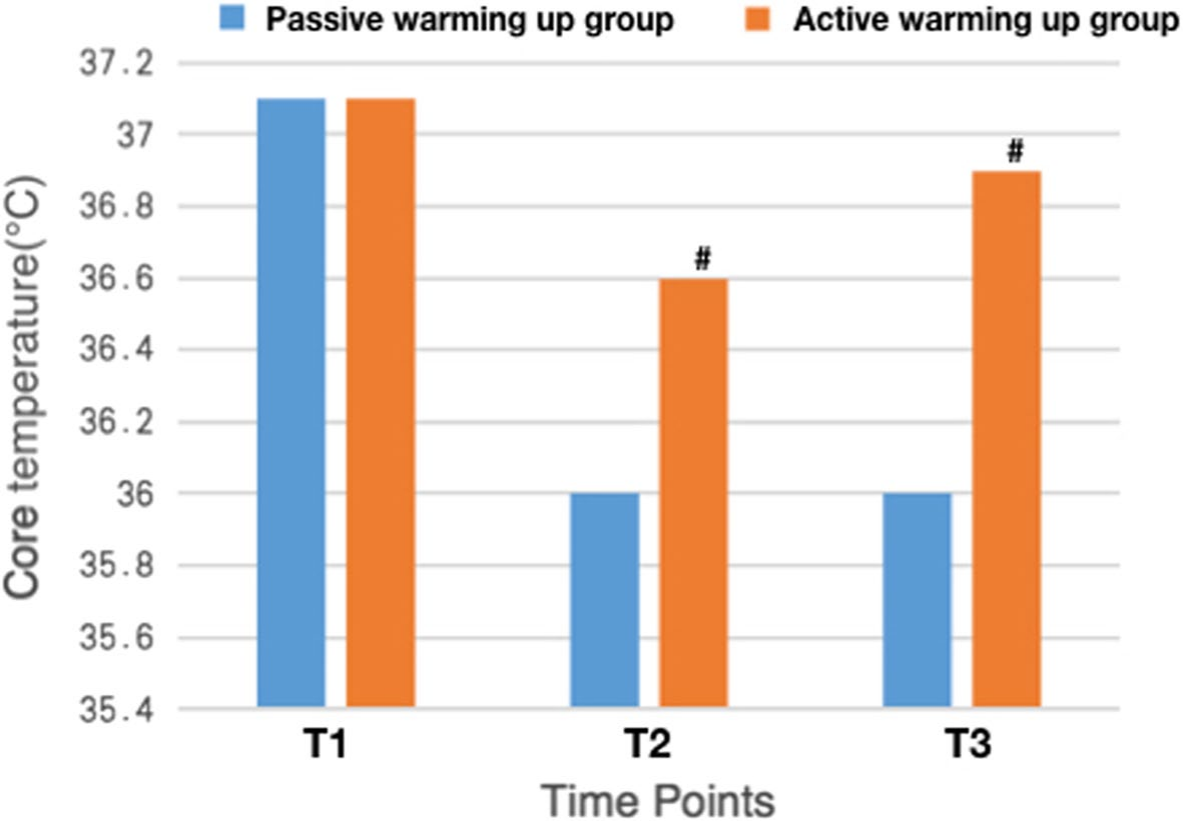
Changes in core body temperatures. Core body temperature in the Control group has been significantly lower than that in the Intervention group since T2 and throughout the surgeries. T1, 5 min before anesthesia induction. T2, 1 h after operation initiation. T3, completion of the surgeries

In both groups, core body temperature was 37.1 ± 0.3 °C at T1 (*p* > 0.05). The temperature descended with a prolonged duration of anesthesia, witnessing a steeper descending slope in the Control group. Core body temperature in the Control group has been significantly lower than that in the Intervention group since 1 h after operation initiation (T2, 36.0 ± 0.5 °C vs. 36.6 ± 0.4 °C, *p* < 0.05, Fig. 2) and throughout till the completion of surgeries (T3, 36.0 ± 0.8 °C vs. 36.9 ± 0.5 °C, *p* < 0.05, Fig. 2).

### Standard coagulation tests

No significant difference was detected in standard coagulation tests (PT, APTT, INR, FBG, PLT) between Control and Intervention groups at surgical closure (see Fig. 2).

### Thromboelastography (TEG) parameters

#### Control vs. Intervention groups at surgical closure

**Fig. 3:**
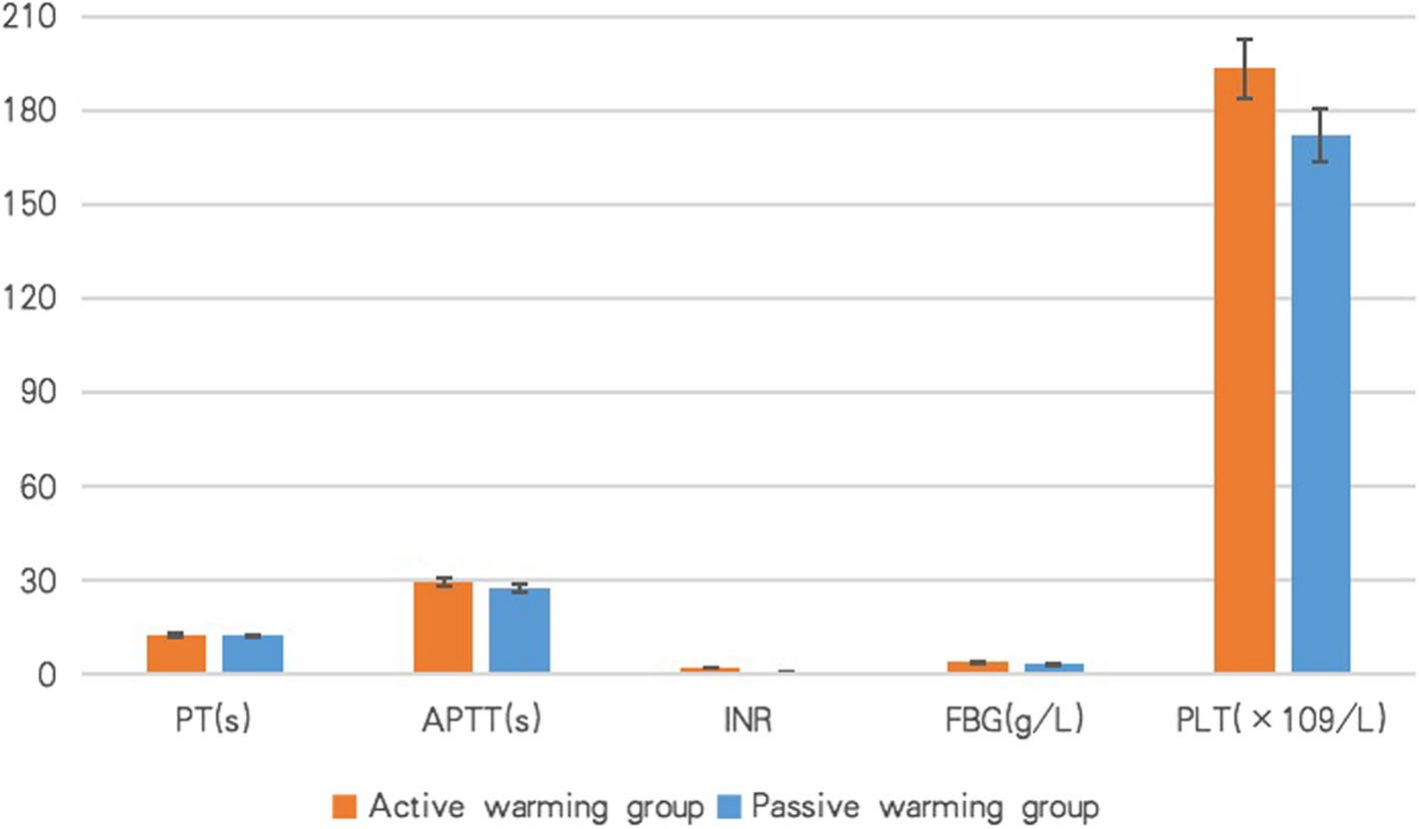
No significant difference in PT, APTT, INR, FBG level, or PLT count was detected between active warming (Intervention) group and passive warming (Control) group at surgical closure

ACT and K time in the Control group were significantly prolonged compared with those in the Intervention group (122.2 ± 19.3 s vs. 117.3 ± 15.2 s for ACT, *p* = 0.01, 1.9 ± 1.0 min vs. 1.3 ± 0.4 min for K time, *p* = 0.02, Fig. 3). Significantly, higher values of MA and α angle were detected in the Intervention group than in the Control group (64.2 ± 5.7 mm vs. 59.4 ± 4.5 mm for MA, *p* = 0.04, 74.9 ± 4.4° vs. 70.4 ± 5.2° for α angle, *p* = 0.05, Fig. 3). No significant difference was detected in LY30 between groups (0.3 ± 0.6 vs 0.6 ± 0.8, *p* = 0.83).

### TEG parameters tested at 37 °C vs. at real core temperatures.

The ACT and K times were significantly prolonged when TEG tests were adjusted to the actual core temperature from the Control group (128.1 ± 8.1 s vs. 122.2 ± 19.3 s for ACT, *p* = 0.01, 2.4 ± 1.4 min vs. 1.9 ± 1.0 min for K time, *p* = 0.04, Fig. 3). The MA and *α* angle values were significantly reduced as compared to the parameters tested at 37 °C (57.0 ± 9.9 mm vs. 59.4 ± 4.5 mm for MA, *p* = 0.03, 67.4 ± 6.3° vs 70.4 ± 5.2° for α angle, *p* = 0.04, Fig. 3). No significant difference was detected in LY30 based on test temperatures (0.3 ± 0.6 vs 0.4 ± 0.7, *p* = 0.12).

### *Postoperative* s*hivering and* w*aking* t*ime*

**Fig. 4:**
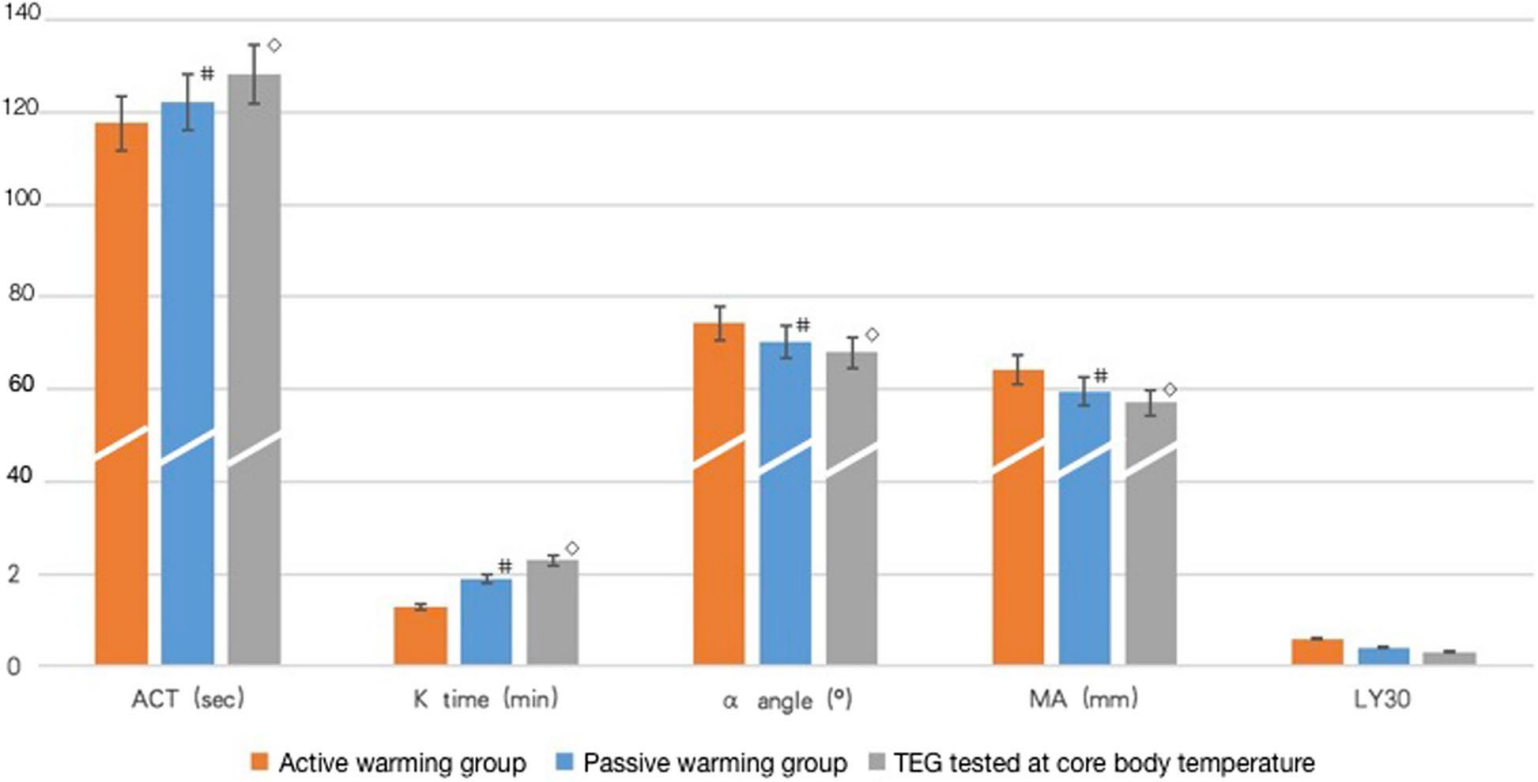
Thromboelastography (TEG) parameters compared between groups at surgical closure. #*p* < 0.05 as compared with active warming group (Intervention group); ♢*p* < 0.05 as compared with passive warming group (Control group) tested at 37 °C

Significantly increased cases of postoperative shivering were observed in the Control group than in the Intervention group (6/32 vs. 1/32, *p* = 0.04, Fig. 4). Waking time was significantly prolonged in the Control group than in the Intervention group (1.0 ± 0.4 h vs. 0.7 ± 0.2 h, *p* = 0.03, Fig. 4).

## Discussion

This is the first study conducted to detect the effect of mild core hypothermia on coagulation functions using both standard coagulation test and TEG in thoracic surgeries. Although no differential hemostatic disturbance was detected using the standard coagulation test, TEG revealed that mild hypothermia significantly exacerbated coagulation disturbances by decreasing MA and *α* angle and prolonging ACT and K time. Furthermore, TEGs conducted under core body temperatures revealed more impaired coagulation than tests incubated at 37 °C.

Hemostatic dysfunction under mild hypothermia was revealed using TEG parameters in this study. Prolonged ACT and K time indicated lower efficiency in attaining clot formation and reaching 20 mm of clot strength, while decreased *α* angles and MA values indicated delayed clot strengthening by fibrin build-up and reduced maximum strength of clot (Othman and Kaur 2017; Thromboelastography: a practice summary for nurse practitioners treating hemorrhage. 2015). In supporting these clinical findings, laboratory research revealed that mild hypothermia induced platelet dysfunction and fibrinogen dysregulation in vitro (Poucke et al. 2014; Kermode et al. 1999; Itenov et al. 2023; Trąbka-Zawicki et al. 2019; Kander and Schött 2019). The superimposed derangements of clot structure and fibrin networks may lead to excessive blood loss in surgical patients receiving passive warming, as observed in our previous clinical investigation and others (Schmied et al. 1996; Yi et al. 2018).

However, no significant difference was detected in intraoperative blood losses between the two groups in this study. It may be attributable to the fact that the operations in both groups were performed by the same team of surgeons, which reduced the heterogeneity introduced by different surgical practitioners. It is also noteworthy that the non-differential hemorrhage amount has eliminated the confounding effect of blood loss on hemostatic test results, since blood losses have been reported to either accelerate or impair coagulation system (Zaar et al. 2014; Davenport and Brohi 2016). Therefore, the hemostatic impairment and hemorrhagic tendency under mild hypothermia were revealed independent of blood losses in this study.

According to our results, the fibrinolytic process appeared unimpacted despite the hemostatic impairment. LY30 represents the percent change in clot strength at 30 min, reflecting a process less dependent on initial enzymatic activity. The comparable LY30 values in both groups indicated that the fibrinolytic process stayed undisturbed under mild hypothermia. The unaffected fibrinolytic process inspired an assumption that antifibrinolytic agents should not be provided as the first-line treatment of hypothermia coagulopathy in major thoracic surgeries. Constituent transfusion may be more efficacious (Durila et al. 2015) and more secure in correcting the cross-links of the coagulation cascade, considering the thrombotic risks carried by antifibrinolytic treatments (Hoyt et al. 2021).

In this study, standard laboratory coagulation tests failed to describe the hemostatic disturbances, while TEG revealed quantifiable differences in coagulation status between the Intervention and Control groups. The routinely used standard tests are conducted on platelet-poor plasma samples warmed to 37 °C (Shimokawa et al. 2003; Haas et al. 2012^)^, failing to evaluate the cellular interactions of clotting. In contrast, TEG measures the global viscoelastic changes associated with the coagulation cascade, the dynamic interaction of fibrin polymerization and platelets, the final clot structure, and the fibrinolytic process (Burton and Jandrey 2020; Wikkelsø et al. 2017). The test includes more steps of the coagulation cascade than PT and APTT tests and is therefore more sensitive to the cumulative slowing of the enzymatic reactions based on temperature changes. Resultingly, TEG calibrated to hypothermic core temperature (33–35 °C) revealed more impaired hypothermic coagulopathy than TEGs under 37 °C in this study, which has also been reported in previous studies (Forman et al. 2014; Nitschke et al. 2021). Therefore, temperature-adjusted TEGs may provide a preferable method of hemostatic monitoring and transfusion guidance in patients undergoing mild hypothermia, which warrants further clinical investigations.

**Fig. 5:**
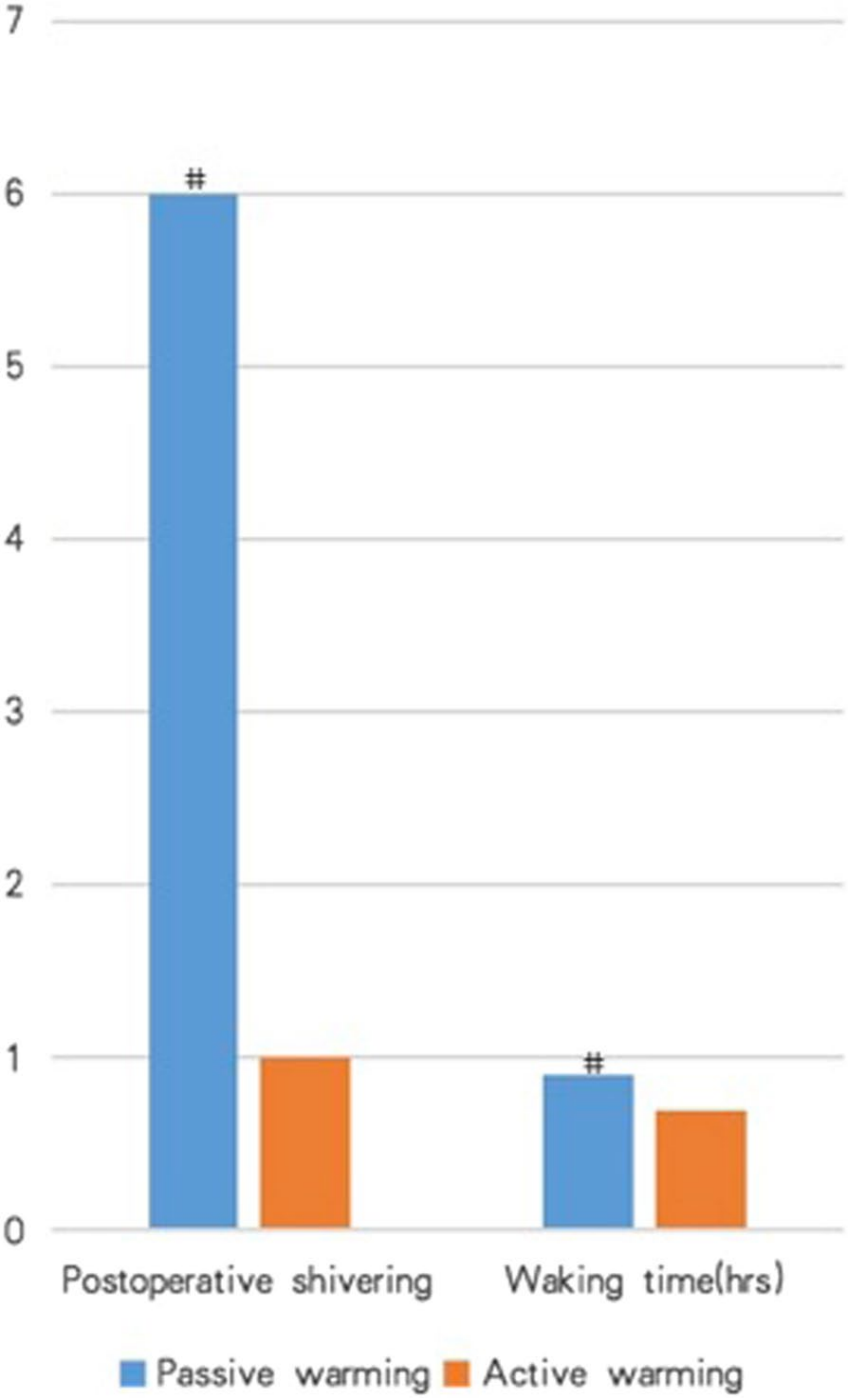
Postoperative shivering occurrence and waking time were significantly increased in passive warming (Control) group as compared with active warming (Intervention) group. #*p* < 0.05 as compared with active warming group (Intervention group)

Mild hypothermia increases postoperative shivering to approximately 40% of unwarmed patients recovering from general anesthesia (Witte and of DSTJOTAS 2002). The shivering is associated with substantial discomfort and a number of potentially deleterious sequelae, including increased oxygen consumption and carbon dioxide production (Sessler 2009), adrenergic activation (Frank et al. 1995), increased cardiac output, tachycardia, and hypertension (Frank et al. 1995). Besides, mild hypothermia also decreases the metabolism of most anesthetics (Vitez et al. 1974; Leslie et al. 1995). The additive effects of the postoperative shivering and decreased drug metabolism inevitably prolong patients’ recovery after surgeries, as was observed in this study and others (Lenhardt et al. 1997). However, whether the maintenance of core normothermia reduces the shivering occurrence and postoperative recovery time still awaits confirmation by larger-scaled randomized studies in the future (Fig. 5).

## Conclusion

Hemostatic impairment was revealed using temperature-based TEG tests in patients receiving thoracic surgeries under mild hypothermia. Standard coagulation tests (PT and APTT) failed to quantify the coagulation disturbance, rendering TEG a potential method of hemostatic monitoring and transfusion guidance in patients undergoing mild hypothermia.

## Data Availability

The datasets generated and/or analyzed during the current study are available from the corresponding author on reasonable request.

## References

[CR1] Alparslan V, Kus A, Hosten T (2018). Comparison of forced-air warming systems in prevention of intraoperative hypothermia. J Clin Monit Comput.

[CR2] Burton AG, Jandrey KE (2020). Use of thromboelastography in clinical practice. Vet Clin North Am Small Anim Pract.

[CR3] Davenport RA, Brohi K (2016). Cause of trauma-induced coagulopathy. Curr Opin Anaesthesiol.

[CR4] Durila M, Lukáš P, Astraverkhava M, Vymazal T (2015). Evaluation of fibrinogen concentrates and prothrombin complex concentrates on coagulation changes in a hypothermic in vitro model using thromboelastometry and thromboelastography. Scand J Clin Lab Invest.

[CR5] Forman KR, Wong E, Gallagher M, McCarter R, Luban NLC, Massaro AN (2014). Effect of temperature on thromboelastography and implications for clinical use in newborns undergoing therapeutic hypothermia. Pediatr Res.

[CR6] Frank SM, Higgins MS, the MBTJO, 1995. The catecholamine, cortisol, and hemodynamic responses to mild perioperative hypothermia a randomized clinical trial. pubsasahqorg. 10.1097/00000542-199501000-00012. 10.1097/00000542-199501000-00012 7832339

[CR7] Haas T, Spielmann N, Mauch J (2012). Comparison of thromboelastometry (ROTEM®) with standard plasmatic coagulation testing in paediatric surgery. Br J Anaesth.

[CR8] Hoyt BW, Baird MD, Schobel S (2021). Tranexamic acid administration and pulmonary embolism in combat casualties with orthopaedic injuries. OTA Int.

[CR9] Itenov TS, Kromann ME, Ostrowski SR (2023). Mild induced hypothermia and coagulation and platelet function in patients with septic shock: secondary outcome of a randomized trial. Acta Anaesthesiol Scand.

[CR10] Kander T, Schött U (2019). Effect of hypothermia on haemostasis and bleeding risk: a narrative review. J Int Med Res.

[CR11] Kermode JC, Zheng Q, Blood EM, the TJO, 1999. Marked temperature dependence of the platelet calcium signal induced by human von Willebrand factor. ashpublicationsorg. 10.1182/blood.V94.1.199.413k14_199_207. 10381514

[CR12] Kim D (2019). Postoperative hypothermia. Acute Crit Care.

[CR13] Kurz A, Sessler DI, Narzt E (1995). Postoperative hemodynamic and thermoregulatory consequences of intraoperative core hypothermia. J Clin Anesth.

[CR14] Lenhardt R, Marker E, Goll V (1997). Mild intraoperative hypothermia prolongs postanesthetic recovery. Anesthesiology.

[CR15] Leslie K, Sessler DI (2003). Perioperative hypothermia in the high-risk surgical patient. Best Pract Res Clin Anaesthesiol.

[CR16] Leslie K, Sessler DI, Bjorksten AR, Moayeri A (1995). Mild hypothermia alters propofol pharmacokinetics and increases the duration of action of atracurium. Anesth Analg.

[CR17] Matika R, Ibrahim M, Patwardhan A (2017). The importance of body temperature: an anesthesiologist’s perspective. Temperature (austin).

[CR18] Mori S, Noda Y, Tsukamoto Y (2019). Perioperative outcomes of thoracoscopic lung resection requiring a long operative time. Interact Cardiovasc Thorac Surg.

[CR19] Nitschke T, Groene P, Acevedo A-C, Kammerer T, Schäfer ST (2021). Coagulation under mild hypothermia assessed by thromboelastometry. Transfus Med Hemother.

[CR20] Othman M, Kaur H (2017). Thromboelastography (TEG). Hemostasis and Thrombosis.

[CR21] Polderman KH (2012). Hypothermia and coagulation. Crit Care.

[CR22] Ruzicka J, Stengl M, Bolek L, Benes J, Matejovic M, Krouzecky A (2012). Hypothermic anticoagulation: testing individual responses to graded severe hypothermia with thromboelastography. Blood Coagul Fibrinolysis.

[CR23] Schmied H, Kurz A, Sessler DI, Kozek S, Reiter A (1996). Mild hypothermia increases blood loss and transfusion requirements during total hip arthroplasty. Lancet.

[CR24] Sessler DI. Mild perioperative hypothermia. August 2009. 10.1056/NEJM199706123362407.

[CR25] Shimokawa M, Kitaguchi K, Kawaguchi M, Sakamoto T, Kakimoto M, Furuya H (2003). The influence of induced hypothermia for hemostatic function on temperature-adjusted measurements in rabbits. Anesth Analg.

[CR26] Thromboelastography: a practice summary for nurse practitioners treating hemorrhage. J Nurse Pract. 2015;11(7):702–709. 10.1016/j.nurpra.2015.05.006. 10.1016/j.nurpra.2015.05.006 PMC4528385 26273234

[CR27] Trąbka-Zawicki A, Tomala M, Zeliaś A (2019). Adaptation of global hemostasis to therapeutic hypothermia in patients with out-of-hospital cardiac arrest: thromboelastography study. Cardiol J.

[CR28] Van Poucke S, Stevens K, Marcus AE, Lancé M (2014). Hypothermia: effects on platelet function and hemostasis. Thromb J.

[CR29] Vitez TS, White PF, of EETJOTAS, 1974. Effects of hypothermia on halothane MAC and isoflurane MAC in the rat. 10.1097/00000542-197407000-00020. 10.1097/00000542-197407000-00020 4151813

[CR30] Wikkelsø A, Wetterslev J, Møller AM, Afshari A (2017). Thromboelastography (TEG) or rotational thromboelastometry (ROTEM) to monitor haemostatic treatment in bleeding patients: a systematic review with meta-analysis and trial sequential analysis. Anaesthesia.

[CR31] De Witte J, of DSTJOTAS, 2002. Perioperative shivering: physiology and pharmacology. pubsasahqorg. 10.1097/00000542-200202000-00036. 10.1097/00000542-200202000-00036 11818783

[CR32] Yi J, Lei Y, Xu S (2017). Intraoperative hypothermia and its clinical outcomes in patients undergoing general anesthesia: national study in China. PLoS ONE.

[CR33] Yi J, Liang H, Song R, Xia H, Huang Y (2018). Maintaining intraoperative normothermia reduces blood loss in patients undergoing major operations: a pilot randomized controlled clinical trial. BMC Anesthesiol.

[CR34] Yi J, Xiang Z, Deng X (2015). Incidence of inadvertent intraoperative hypothermia and its risk factors in patients undergoing general anesthesia in Beijing: a prospective regional survey. PLoS ONE.

[CR35] Zaar M, Mørkeberg J, Pott FC, Johansson PI, Secher NH (2014). Coagulation competence and fluid recruitment after moderate blood loss in young men. Blood Coagul Fibrinolysis.

